# Loss of Coral Trait Diversity and Impacts on Reef Fish Assemblages on Recovering Reefs

**DOI:** 10.1002/ece3.70510

**Published:** 2024-10-30

**Authors:** Lintao Huang, Mike McWilliam, Chengyue Liu, Xiaolei Yu, Lei Jiang, Chen Zhang, Yong Luo, Jianhui Yang, Xiangcheng Yuan, Jiansheng Lian, Hui Huang

**Affiliations:** ^1^ CAS Key Laboratory of Tropical Marine Bio‐Resources and Ecology; Guangdong Provincial Key Laboratory of Applied Marine Biology, South China Sea Institute of Oceanology Chinese Academy of Sciences Guangzhou China; ^2^ University of Chinese Academy of Sciences Beijing China; ^3^ Hawaii Institute of Marine Biology University of Hawaii Manoa Kaneohe Hawaii USA; ^4^ Centre for Biological Diversity, Scottish Oceans Institute University of St Andrews St Andrews UK; ^5^ Southern Marine Science and Engineering Guangdong Laboratory (Guangzhou) Guangzhou China; ^6^ CAS‐HKUST Sanya Joint Laboratory of Marine Science Research Key Laboratory of Tropical Marine Biotechnology of Hainan Province Sanya China; ^7^ Sanya National Marine Ecosystem Research Station; Tropical Marine Biological Research Station in Hainan Chinese Academy of Sciences Sanya China

**Keywords:** coral reefs, ecological succession, functional traits, marine protected area, reef fish, reef‐building coral

## Abstract

Understanding patterns of biodiversity change is essential as coral reefs experience recurrent cycles of disturbance and recovery. Shifts in the total cover and species composition of habitat‐forming corals can have far‐reaching consequences, including shifts in coral functional traits and impacts on local fish assemblages. We surveyed coral and fish assemblages along the southern coast of Hainan Island near Sanya, China, in 2006, 2010, and 2018, during a period with repeated mass bleaching events. We showed that coral biodiversity in this region is in a state of flux, with losses and gains in coral cover and an increase in species richness over time. Despite increasing species diversity, the region suffered a loss of coral trait diversity by 2010, with an incomplete recovery by 2018, owing to declines in species with key habitat‐forming traits (e.g., high surface areas and fractal structure) such as corymbose corals. Concurrently, there was an increase in functional redundancy due to the proliferation of the dominant encrusting and massive corals. Coral cover was positively associated with the abundance of reef fish, indicating that the changes observed in coral abundance can impact reef‐associated species. These results demonstrate that the slow recovery of coral biodiversity in southern Hainan Island has been hampered by the loss of specific coral traits and highlight the importance of protecting vulnerable coral traits in conservation and management strategies.

## Graphical abstract

Analyzing 12 years of field survey data, our study uncovers asynchronous changes in species richness and trait diversity during the recovery of reef‐building coral communities on southern Hainan Island. The decrease in corymbose branching corals and the rise in functionally redundant corals explain the loss of trait diversity during coral recovery. Moreover, a positive association between coral cover and the abundance of reef fish suggests potential impacts on reef‐associated species, indicating that the changes observed in coral abundance may impact reef‐associated species.

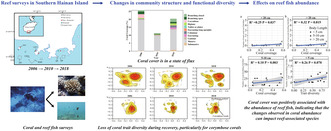

## 1 Introduction

Global climate change and localized human activities are altering the numbers and types of species within ecosystems (Hooper et al. 2012; Johnson et al. 2017). Increasing disturbances, such as storms, wildfires, or mass coral bleaching, mean that many degraded ecosystems are in a state of recovery or secondary succession (Acevedo‐Charry and Aide 2019; McWilliam et al. 2020; Coradini, Krejčová, and Frouz 2022). These disturbances often prevent degraded ecosystems from completely returning to their previous state (Prach and Walker 2019; Coradini, Krejčová, and Frouz 2022). Understanding the dynamics of secondary succession during community recovery is essential for understanding biodiversity changes in heavily disturbed areas and will ultimately help in generating ecosystem management plans and conservation strategies. Specifically, identifying and targeting lost species or traits in degraded communities, as well as missing species or traits during recovery, can be crucial for biodiversity conservation efforts (Hoey and Bellwood 2009; Acevedo‐Charry and Aide 2019; McLean et al. 2019).

Coral reef ecosystems are highly biodiverse, with over a quarter of marine fish species residing in reef habitats (Fisher et al. 2015). These ecosystems are highly valued for their ecological and economic importance (Woodhead et al. 2019). However, coral reef ecosystems are experiencing severe degradation owing to a range of chronic and acute impacts (Hughes et al. 2017, 2018; Heery et al. 2018). Recent research has revealed that the degradation of reefs is not only characterized by a decline in reef‐building coral cover but also by a simplification of the structure of the coral communities and a decline in reef biodiversity (Hughes, Huang, and Young 2013; Cybulski et al. 2020). Many of the changes in coral species composition can be attributed to a disruption to the natural cycles of disturbance and recovery of coral reefs (McWilliam et al. 2020).

Reef‐building coral cover is the most commonly used indicator for a coral reef's status (John, Zandy, and Caroline 2000; Darling et al. 2019). In addition, many studies have analyzed reef biodiversity change by examining changes in taxonomic diversity through metrics such as species richness (Richards et al. 2008), population demographics (Williams and Miller 2012), and species composition (McClanahan 2014; Jouval et al. 2020; Mizerek et al. 2021). Following an expansion of data and tools available to analyze trait diversity, increased attention has also been given to morphological, physiological, and behavioral trait changes during community succession (Mouillot et al. 2013; Bellwood et al. 2019). Species and trait diversity can change in different ways along successional gradients (Acevedo‐Charry and Aide 2019) and can provide complementary indicators of how reef biodiversity is changing (McWilliam et al. 2020; Sommer, Butler, and Pandolfi 2021). As the primary framework‐builders in reef ecosystems, corals influence the community structure of associated reef fauna through the production of complex three‐dimensional habitats (Lazarus and Belmaker 2021). Changes in the trait diversity of corals can impact reef fish assemblages through a variety of mechanisms, including declines in three‐dimensional refuge space, shifts in feeding relationships, or the loss of coral traits that facilitate adaption to diverse reef habitats (Darling et al. 2017).

Coral reefs in the South China Sea are facing multiple pressures and have experienced severe degradation that has been well‐documented since the 1990s (Lian, Huang, and Huang 2010; Hughes, Huang, and Young 2013; Yu et al. 2019). Compared to offshore reefs in the South China Sea, nearshore reefs have been drastically affected by human activities such as coastal construction, overfishing, and increasing terrigenous input into the coastal seas (Hughes, Huang, and Young 2013). Improved management and protection of coral reefs in China have stabilized the coral status, with some areas showing coral cover recovery in recent years, including southern Hainan Island (Huang, Chen, and Huang 2021; Sun et al. 2022), providing valuable research opportunities to analyze the recovery process of coral assemblages. We analyzed the species diversity and trait diversity of corals along the southern coast of Hainan Island, combining field surveys and trait analyses to explore coral assemblages' succession and their impacts on local reef fish assemblages. The findings can provide insights into the trait changes and successional patterns of coral assemblages following degradation and recovery.

## 2 Materials and Methods

### 2.1 Description of Study Area

**FIGURE 1:**
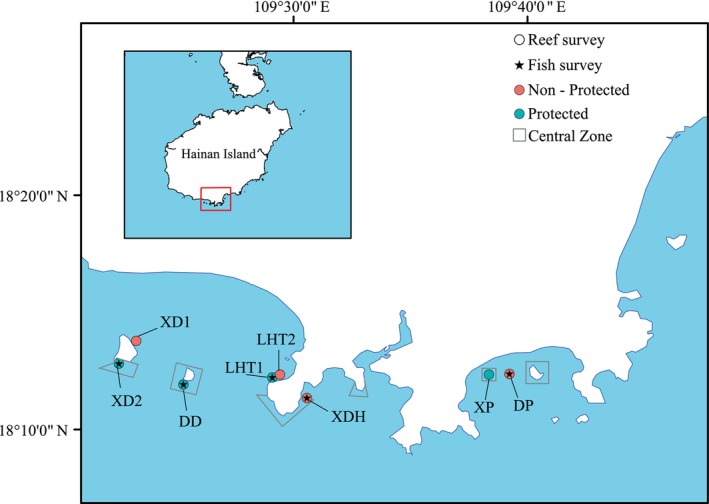
Location of the eight coral reef survey sites. Circles indicate coral reef benthos and substrate surveys sites, and asterisks indicate reef fish surveys sites green for protected reef areas and orange for unprotected reef areas. The gray boxed line represented the central zone. Xidao Island (XD1), southwest of Xidao Island (XD2), south of Dongdao Island (DD), south Luhuitou (LHT1), north Luhuitou (LHT2), Xiaodonghai (XDH), Dongpai Island (DP), and Xipai Island (XP).

The coral reefs in southern Hainan Island (18°12′ N—18°14′ N, 109°21′ E—109°38′ E) are a vital component of the northern South China Sea's marine ecosystem, home to over 180 species of reef‐building corals (Huang, Chen, and Huang 2021). However, the coral reefs here are faced with various threats arising from human activities and global warming (Lian, Huang, and Huang 2010; Li et al. 2012). For instance, the region was negatively impacted by coastal construction until 2010 (Hughes, Huang, and Young 2013) and then experienced coral bleaching in 2010, 2015, and 2017 (Li et al. 2012; Huang, Chen, and Huang 2021). These challenges have left the coral assemblages in the region in a degraded state (Zhao et al. 2012; Huang, Chen, and Huang 2021). To track changes in coral and fish assemblages here, eight fringing reef areas with similar abiotic conditions in southern Hainan Island were selected for this study (Figure 1). Previous studies have shown that reefs in southern Hainan Island were already degraded in 2006 (Lian, Huang, and Huang 2010). With the addition of conservation measures such as prohibition of human activities in coral reef areas and legal protection of reef‐building coral species in recent years (Sun et al. 2022), reefs in southern Hainan Island appear to be recovering. The time series data selected for this study were 2006, 2010, and 2018, covering recent stages of degradation and recovery of reef‐building corals.

To further explore the impact of protected areas on the recovery of coral assemblages, we categorized the conservation status of eight coral reef survey sites within the Sanya Coral Reef National Nature Reserve in Hainan. The Reserve’ Central Zones, which prohibit all human activities, were considered protected, while its experimental zones, which allow tourism but prohibit fishing, did not meet the criteria for protected in this study (Huang et al. 2017). Consequently, three sites (XD2, DD, and XP) in the Central Zones were categorized as protected. An additional site, south Luhuitou (LHT1), was also classified as protected as it is managed by the Tropical Marine Biological Research Station, which enforces a ban on all human activities. In total, four sites were designated as protected, and the remaining four as unprotected (Figure 1).

### 2.2 Reef Benthos and Substrate Composition Surveys

We used the Line Intercept Transect method to monitor benthic and substrate composition, sampling at a depth of 3–6 m (English, Wilkinson, and Baker 1997). Different years were surveyed in the same position according to latitude and longitude coordinates. The 50‐m transects were used in 2006 for each site depth, and six 10‐m repeated transects were used for each site depth in subsequent survey years (Table S1). To study the changes in the composition of reef benthos and substrates, the abundance of 12 taxa and substrates, including rock, bare reef, reef‐building coral, Crude coralline algae (CCA), sand, soft corals, zoantharian, corallimorpharian, Spores, sea anemones, macroalgae (MA), and others, was quantified. Among them, bare reefs represented dead coral covered by sediment or turf algae. We used linear mixed models to test the effects of marine protected areas (MPAs) and survey years on the variation of reef‐building coral cover. We included the random effect of the site to account for variation among sites. Fixed effect parameters were tested using Wald tests with Satterthwaite's method.

### 2.3 Coral Species Richness and Assemblages' Composition Surveys

Reef‐building corals were identified to species level where possible, except for some (e.g., *Acropora*, *Montipora*, *Porites*, and *Goniopora*) that were identified to genus. We clarified that the term “coral” was used as “reef‐building coral” hereafter to avoid ambiguity caused by the presence of “soft coral.” Coral species richness data were obtained by counting the numbers of species on each transect, including taxa identified to genus (possibly leading to an underestimation of species richness). To account for variations in sampling methods across years, we employed a rarefaction analysis using the *iNext* function to compute sample coverage and estimate species diversity for each coral assemblage (Hsieh, Ma, and Chao 2016). Briefly, we calculated Hill numbers at different *q* orders: *q* = 0 representing species richness, *q* = 1 representing Shannon diversity, and *q* = 2 representing Simpson diversity. The shift from *q* = 0 to *q* = 2 indicated a diminishing emphasis on the impact of rare species within the index (Chao et al. 2014). Moreover, the typical growth forms of corals were retrieved from the Coral Traits Database (https://coraltraits.org) (Madin, Anderson et al. 2016) to estimate cover across different groups. Considering that genera may have variable growth forms, for those species identified as genus, the growth form with the highest frequency of occurrence within the genus was selected as a representative.

Principal Coordinates Analysis (PCoA) was used to visually assess the differences in coral assemblage structure among years. Furthermore, we used permutational multivariate analysis of variance (PERMANOVA) with 999 permutations to examine spatial and temporal variations in coral species composition. The PERMANOVA model included survey years and protection status as fixed factors together with their interaction effect to test for time and protection effects separately. After that, we conducted a distance‐based redundancy analysis (db‐RDA) (Legendre and Anderson 1999) to identify which types among 12 types of benthos and substrates contribute significantly to differences in coral assemblages' composition. To better screen the effect of benthos or substrate type on coral assemblage structure in the db‐RDA, we performed forward selection and sequentially added benthos or substrate groups that best explained variation in coral assemblage structure based on the Akaike Information Criterion (AIC). The benthos or substrate types combined with the lowest AIC were selected for the final db‐RDA analysis.

### 2.4 Coral Species Traits Data Collection and Functional Trait Diversity Calculation

Since corals are the framework builders of coral reefs, the focus of this study was on functions associated with corals, with a specific focus on habitat‐forming traits (Table S2). Seven functional traits of corals were used to build trait space, including growth rate, skeletal density, colony size, colony height, corallite width, intra‐colony space size, and colony surface area to volume ratio, following previous studies (McWilliam et al. 2018), because those traits were considered to be representative of corals capacity to produce carbonate structures (growth and skeletal density), form a 3D framework (largely based on morphological traits), and nutrient capture (Table S2). Selected traits are also expected to be ecologically valuable for reef fish; the 3D structure built by corals is an important habitat for many fish species; and the skeletal density and growth rate of corals ensure the stability of the habitat structure (Coker, Wilson, and Pratchett 2014; Darling et al. 2017). Species‐level traits of corals are available in the Coral Traits Database (Madin, Anderson et al. 2016). Traits that do not have measured values are populated following the method of Madin, Hoogenboom et al. (2016), including the genus‐level values. Briefly, a regression approach based on coral growth form and phylogenetic information was used to fill in missing data for coral traits. Missing traits were filled in for 11 of the 91 coral species. After that, we placed all trait scores for each coral species into numerical (range: 1–5) categories following McWilliam et al. (2018) (Table S2).

Gower distance matrix‐based PCoA analysis of seven traits was used to construct a trait space for the different coral assemblages. Functional trait diversity was measured as the volume of trait space occupied by each assemblage in four dimensions. Functional redundancy was measured by the sum of distances from five neighboring points, and functional dispersion was measured by the mean abundance‐weight distance in multidimensional trait space of individual species to the abundance‐weight centroid of all species (Laliberté and Legendre 2010). We combine all assemblages in a year across all sites into a single meta‐assemblage to calculate the trait parameters. Meanwhile, ANOVA and LSD post hoc testing were used to compare the site functional trait parameters and species richness at different years.

### 2.5 Reef Fish Abundance, Assemblages' Composition Surveys and Their Relationship to Coral Cover, Coral Species Richness, and Coral Functional Trait Diversity

To monitor the status of the reef fish assemblages in Sanya, the coral reef fish survey was conducted in the XD2, DD, LHT‐1, XDH, and DP. Fish assemblage data were collected using the underwater visual censuses. The 150‐m transects were used in 2006 and 2010 for each site depth, and 60‐m transects were used for each site depth in 2018 (Table S1). Fish within a 2‐m‐wide belt transect were counted and identified as species or genus. We recorded the number of reef fish, identified them with the lowest taxonomic level possible, and simultaneously estimated the body length of the reef fish by classifying them into four groups: < 5 cm, 5–10 cm, 10–20 cm, and > 20 cm (Table S1).

To assess the sampling adequacy of reef fish, we also employed the *iNext* function to compute sample coverage and estimate species diversity for each reef fish assemblage based on the abundance of reef fish species (Hsieh, Ma, and Chao 2016). Reef fish abundance at each study site was expressed in units per 60 m^2^. To study changes in the structure of coral reef fish abundance among years across all sites, we separated fish species that are considered as coral dwellers (CD), as previously described in the literature (Froese and Pauly 2017), and other fish are considered as non‐coral dwellers (NCD) (Fontoura et al. 2019). ANOVA was used to compare the abundance of reef fish at different years and to test for changes in abundance over time. Factors with significant results in ANOVA were further tested using the LSD post hoc testing. To investigate the reef fish assemblage structure variation, db‐RDA was used to identify the contribution of NCD and CD reef fish abundance to overall reef fish assemblage composition. PERMANOVA with 999 permutations was utilized to assess differences in fish assemblage composition across years and between protected and non‐protected areas. The PERMANOVA model also incorporated survey years, protection status, and their interaction as fixed factors, allowing for separate testing of temporal and conservation effects and their interaction effect. We used linear mixed models to test the effects of coral cover and coral functional trait diversity on reef fish abundance. We included site and survey year as the random effects to account for variation among sites and over time. Fixed effect parameters were also tested using Wald tests with Satterthwaite's method.

All statistical analyses were implemented in the software R 3.6.3. The species assemblage structure analysis was obtained by *vegan* (Oksanen et al. 2022) and *ADE4* (Thioulouse et al. 2018) packages. The LSD post hoc test was obtained by *agricolae* (Mendiburu 2019) package. Functional trait diversity, functional dispersion, and functional redundancy were obtained using the *iNext* (Hsieh, Ma, and Chao 2016), *FD* (Lalibert, Legendre, and Shipley 2014), *FNN* (Beygelzimer et al. 2019), *ks* (Duong 2014)*, lme4* (Bates et al. 2015), and *lmerTest* (Kuznetsova, Brockhoff, and Christensen 2017) packages.

## 3 Results

### 3.1 Reef Benthos and Substrate Composition

**FIGURE 2:**
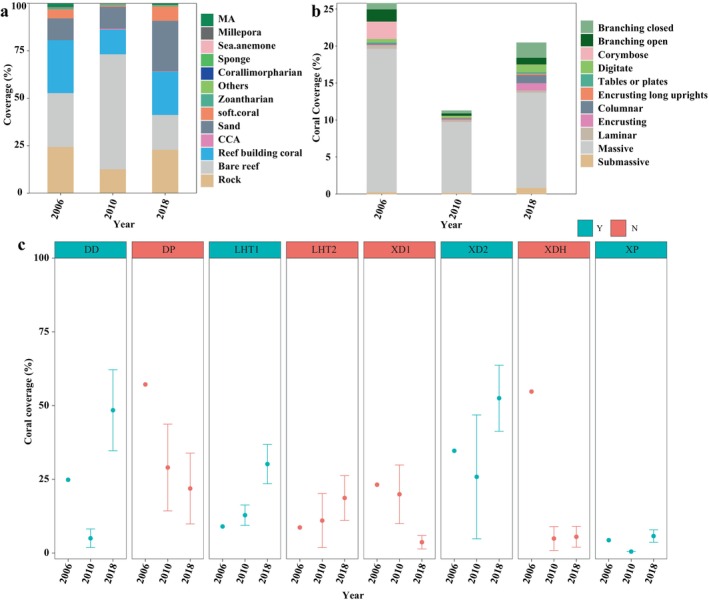
Benthos and substrate composition of coral reefs in southern Hainan Island. (a) Benthos and substrate composition; (b) The average coverage of reef‐building corals with different growth forms across all sites; (c) Coral cover at different survey sites. Note that only one 50 m transect was assessed at each site in 2006. Green for protected reef areas (Y) and orange for unprotected reef areas (N).

From 2006 to 2018, average coral cover across all sites showed a decrease followed by an increase. The average coral cover from all sites was: 27.87% ± 23.79% in 2006; 12.94% ± 14.39% in 2010; and 22.76% ± 21.26% in 2018. The difference in coral cover between 2006 and 2018 was attributed to an increase in soft coral and non‐living substrate. The proportion of corymbose corals represented on reefs showed a visible decline in 2010 and did not increase during the recovery period. The increases in coral cover were mostly among encrusting and submassive coral species (Figure 2b). However, sites have different trends in coral cover variation. Sites of increasing coral cover were DD, LHT1, LHT2, and XD2, and sites of decreasing cover were XD1, DP, and XDH. The coral cover of XP was maintained at a low level (< 5%). The recovery of coral cover (the difference between 2010 and 2018) was significantly higher in protected areas than in non‐protected areas (Figure 2, Table S3).

### 3.2 Coral Species Richness and Assemblages' Composition

**FIGURE 3:**
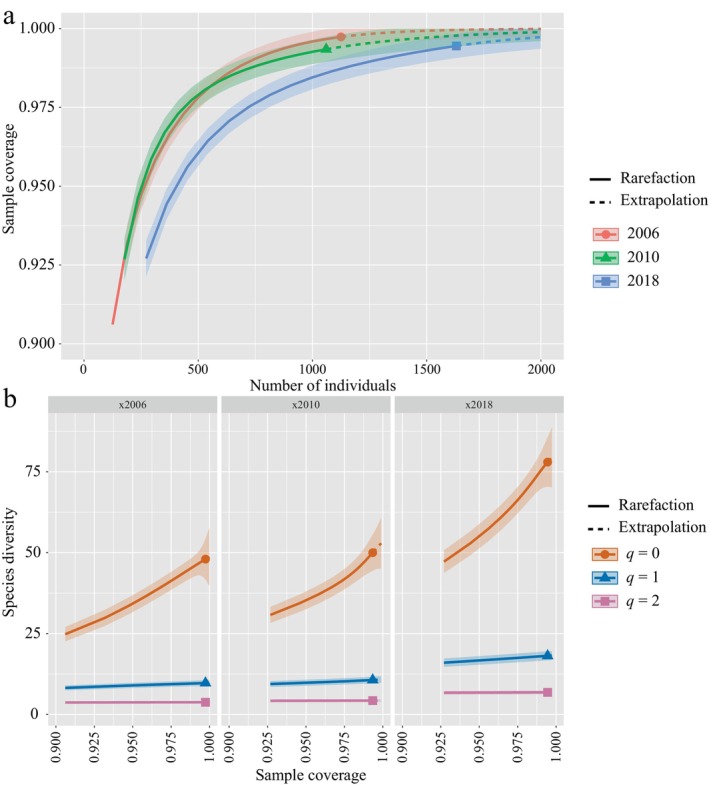
Sample coverage of reef‐building coral assemblages and their species diversity estimation among years. (a) Sample coverage estimation of coral assemblages among years. (b) Coral species diversity estimation of the different *q* orders. When *q* = 0: species richness; *q* = 1: Shannon diversity; *q* = 2: Simpson diversity. The solid shape marks the observed diversity values.

The results showed that the sample coverage of each coral assemblage was greater than 99% despite the different sampling areas of each assemblage, suggesting that the surveys among years were sufficiently representative of the biodiversity of local coral species each year (Figure 3). The coral species richness showed an increasing trend among years, with the number of species in 2018 exceeding the surveys in 2006. The 2006 survey reported 48 species, the 2010 survey reported 50 species, and the 2018 survey reported 78 species (Figure 5a).

**FIGURE 4:**
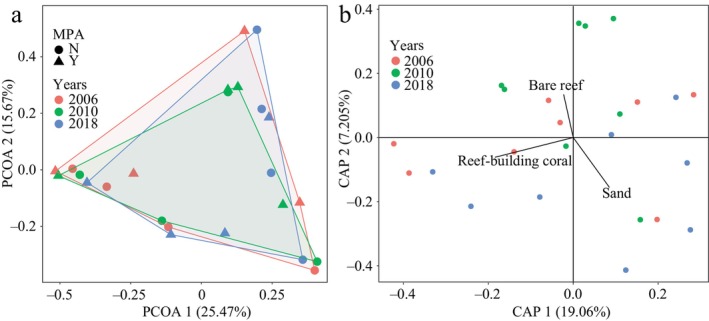
Reef‐building coral assemblages' composition and their relationship with benthos and substrate composition in southern Hainan Island. (a) Principal coordinates analysis (PCoA) on Bray Curtis distances showed the difference in coral assemblage structure among years; color showed survey years; and point shape showed protected status. Y represented protected reef areas and N represented unprotected reef areas; (b) Distance‐based redundancy analysis showed the relationship of coral assemblages with benthos and substrate composition.

According to PERMANOVA, there was no significant difference between 2006, 2010, and 2018 in the composition of coral assemblages (*R* ^2^ = 0.088, *F* test *p*‐value = 0.449) (Figure 4a). This indicated that despite the change in cover, the coral assemblages have not changed significantly in the types of species present and their abundances. Similarly, no significant differences in the composition of coral assemblages were found between protected areas and non‐protected areas (*R* ^2^ = 0.027, *F* test *p*‐value = 0.875) and the interaction between protection status and survey years (*R* ^2^ = 0.077, *F* test *p*‐value = 0.620) (Figure 4a). There were one benthos type and two substrate types that best explained the variation in coral assemblages' composition: coral cover, sand, and bare reef. The coral assemblages in 2010 were primarily structured by bare reefs, consistent with their decline in coral cover (Figure 4b). In contrast, coral assemblages in 2018 were closely associated with sandy substrates, which implies a shift from bare reefs to sandy or rubble substrates during that period.

### 3.3 Functional Trait Parameters

**FIGURE 5:**
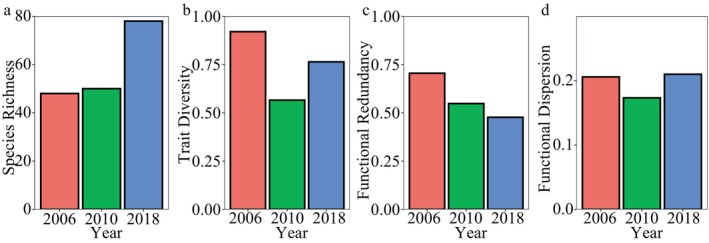
Diversity parameters of reef‐building coral assemblages in southern Hainan Island among years. (a) Species richness; (b) Functional trait diversity; (c) The average sum of nearest neighbor distances for the nearest five species showing the functional redundancy; (d) Functional dispersion. The diversity parameters were calculated by the sum of all survey sites for each year.

**FIGURE 6:**
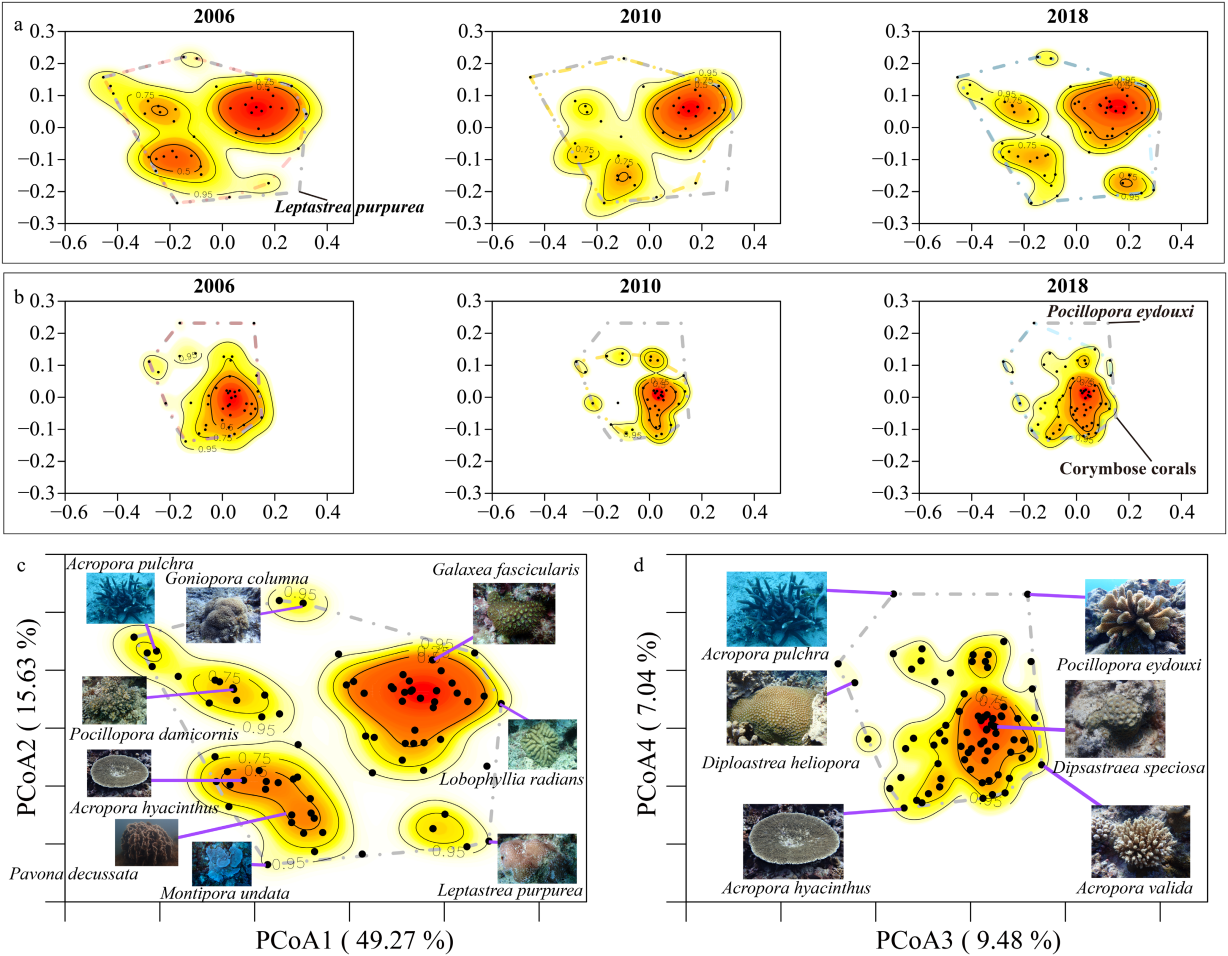
Heat map of species distribution within the trait space for each survey year, with contours indicating peaks of richness and similarity, and the convex hull indicates the size of the trait space, which represented the functional diversity. (a) Principal coordinates component 1 (PCoA1) and PCoA2 of the trait composition of corals among years; (b) PCoA3 and PCoA4 of the trait composition of corals among years; (c) The coral species position in the first two axes of trait space; (d) The coral species position in the 3–4 axes of trait space.

Coral functional trait diversity aggregated across all sites was 92.03% in 2006, 56.57% in 2010, and 76.38% in 2018 (Figure 5b, Figures S1–S3b, Table S4). Functional redundancy, on the other hand, increased over time, as reflected by a reduction in the distance of species from their five neighbors in trait space, 0.71 in 2006; 0.55 in 2010; and 0.48 in 2018 (Figure 5c, Figure S3c, Table S4). Functional dispersion, which reflects the dispersion of species in trait space, showed a decrease followed by an increase, with 0.21 in 2006, 0.17 in 2010, and 0.21 in 2018 (Figure 5d, Figure S3d, Table S4). Further analysis of the trait space revealed that branching species such as *Pocillopora eydouxi* and the corymbose corals (*Acropora valida*, *A. verweyi*, and *A. nasuta*) were missing after 2006 from the survey, which caused strong shifts in trait diversity along PCoA axes 3–4 of trait space (Figure 6, Figure S2). Among them, the abundance of corymbose corals was lower in 2018 (Figures 2b and 6). Moreover, many encrusting and massive corals appeared, such as *Montipora* spp. and *Leptastrea* spp., which modified trait diversity along the first two axes of trait space (Figure 6, Figure S1).

### 3.4 Reef Fish Abundance, assemblies' Composition and Their Relationship to Coral Cover, Coral Species Richness, and Coral Functional Trait Diversity

Fish abundances in all coral reef areas in our analysis decreased in each survey year (Table S5), primarily driven by decreases in non‐coral dwelling (NCD) fish. The total fish abundance decreased from 61.22 ind./60m^2^ in 2006 to 27.39 ind./60m^2^ in 2010, and further decreased to 20.85 ind./60m^2^ in 2018 (Figure S6a). The average abundance of coral‐dwelling (CD) fish was 21.70 ind./60 m^2^ in 2006, 14.75 ind./60 m^2^ in 2010, and 11.10 ind./60 m^2^ in 2018, though variation within years was high and the overall decreasing trend was non‐significant (Figure S6b, Table S5). The abundance of NCD reef fish showed a yearly decrease, from 39.52 ind./60m^2^ in 2006 to 12.64 ind./60m^2^ in 2010 and 9.75 ind./60m^2^ in 2018 (Figure S6c, Table S5).

The rarefaction analysis results also showed that sample coverage of each reef fish assemblage was greater than 97.5%, suggesting that the surveys among years were sufficiently representative of the species diversity of fish species each year (Figure S4). The db‐RDA and PERMANOVA results showed significant changes in reef fish assemblage structure across time (*R* ^2^ = 0.355, *F* test *p*‐value = 0.001), as the fish assemblage in 2006 had more NCD fish than those in 2018 and 2010, while the assemblage structure of CD fish did not vary as much between survey years as NCD fish (Figure S5). We found no significant differences between the reef fish assemblages inside and outside protected reserves (*R* ^2^ = 0.076, *F* test *p*‐value = 0.111) and the interaction between protection status and survey years (*R* ^2^ = 0.128, *F* test *p*‐value = 0.177) (Figure S5).

**FIGURE 7:**
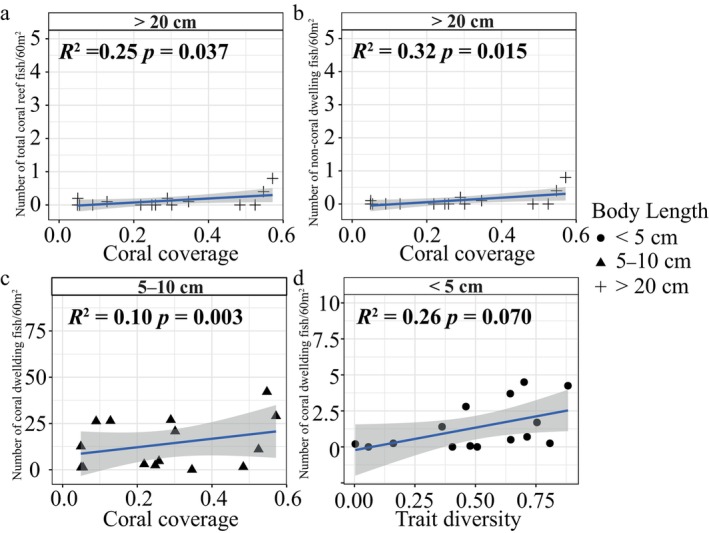
Relationship between reef fish abundance and coral cover or functional trait diversity according to fish body size class with 95% confidence intervals. (a) Regression results for total large (> 20 cm) reef fish abundance and coral cover; (b) Regression results for large (> 20 cm) non‐coral dwelling fish abundance and coral cover; (c) Regression results for 5–10 cm coral dwelling fish abundance and coral cover. (d) Regression results for small coral (< 5 cm) dwelling fish abundance and functional trait diversity. Lines and respective shadows indicate confidence intervals of respective models. *R* ^2^ values represent the results of linear models with coral cover or functional trait diversity as a predictor. *p*‐values indicate the results of the corresponding linear mixed model tests. Only the results of the linear mixed model with *p* < 0.1 were shown here, and other results are presented in Table S3.

The results showed a significant correlation between coral cover and the total abundance of fish larger than 20 cm in body length (Figure 7a, Table S3). There was a significant correlation between coral cover and the total abundance of fish > 20 cm in body length (Figure 7b, Table S3), as well as between coral cover and the abundance of 5–10 cm body length CD fish (Figure 7c, Table S3), indicating that the higher coral cover was associated with greater numbers of large NCD fish and small CD fish. It should be emphasized that many CD fish are small, usually less than 10 cm in length. Despite not being significant, our analysis also revealed a marginally positive correlation (*p* value = 0.070) between coral functional trait diversity and the abundance of small CD fish (< 5 cm), indicating that a higher diversity of traits in the coral assemblage facilitated a greater abundance of small fish (Figure 7d, Table S3).

## 4 Discussion

### 4.1 The Degradation and Recovery of Reef‐Building Corals in Southern Hainan Island

Reef degradation is a pressing issue that affects coral reefs globally (Cruz et al. 2018; Cramer et al. 2021), especially in East and Southeast Asia (Hughes, Huang, and Young 2013; Heery et al. 2018). Since the 1960s, coral cover in southern Hainan Island has plummeted from 80%–90% to 11% due to coastal construction, overfishing, and sedimentation (Yu and Zou 1996; Hughes, Huang, and Young 2013; Huang, Chen, and Huang 2021). Our study revealed fluctuations in coral cover among different sites over the years, with an overall declining and then recovering trend between 2006 and 2018, despite coral bleaching events in 2010, 2015, and 2017 (Li et al. 2012; Huang, Chen, and Huang 2021). This suggests a slow recovery of coral cover in recent years despite ongoing disturbances caused by global warming, which may be related to recent management measures to protect coral reefs, including the prohibition of human activities in coral reef areas and the legal protection of reef‐building coral species (Sun et al. 2022). However, coral cover in southern Hainan Island has not fully recovered to its previous state, particularly for branching coral cover. This contrasts with other areas such as the Great Barrier Reef, South Central Pacific, and Madagascar, where branching coral cover can recover within 10 years (Pratchett, McWilliam, and Riegl 2020; Vessaz et al. 2022; Morais et al. 2023). The slower recovery of branching coral cover in southern Hainan may be due to environmental variations such as an increase in the sand substrate and high turbidity, which may have restricted the habitat of branching corals and suppressed their recovery (Luo et al. 2022, 2023).

The degradation of coral assemblages manifests in a number of ways, not only including a decrease in coral cover but also in the alteration of coral assemblage composition (Hughes, Huang, and Young 2013; Cruz et al. 2018). A shift in the community from structurally complex species (e.g., branching species) to less structurally complex species such as massive, sub‐massive, and encrusting species happened in recent decades globally (Cramer et al. 2020; Cybulski et al. 2020), and each substantially affecting reef habitat quality and three‐dimensional complexity. Here in southern Hainan Island, the coral assemblages have also changed from being dominated by branching *Acropora* spp. to being dominated by massive corals in just a few decades (Yu and Zou 1996; Zhao et al. 2012). The present study showed that the coral assemblages remained dominated by massive corals from 2006 to 2018, indicating that the coral assemblage composition has not recovered to its previous state.

Marine protected areas (MPAs) are increasingly implemented to protect coral reef biodiversity (Mellin et al. 2016). However, more evidence is still needed to understand the effectiveness of MPAs in protecting corals. Some studies suggested that MPAs have little or no impact on coral reef habitats (Toth et al. 2014), while others report positive effects on the protection of coral species (Selig and Bruno 2010; Mellin et al. 2016; Strain et al. 2019). In particular, enhanced, no‐take, and old (> 10 years) MPAs can significantly increase coral cover (Strain et al. 2019). The present study showed that the subsequent recovery of coral cover in the protected habitats was faster than in adjacent unprotected habitats, demonstrating the effectiveness of coral protection by prohibiting human activities in MPA. However, we found that the return of cover did not translate into a return in assemblage composition, implying that MPAs alone are not enough to help the recovery of degraded coral assemblages.

### 4.2 Trait Losses in the Recovering Coral Cover and Species Richness

The species diversity of different fauna can exhibit diverse patterns during ecosystem degradation and recovery (Tölgyesi et al. 2019; Li et al. 2021; Isingoma et al. 2023). In the current study, despite fluctuating coral cover, coral species diversity gradually increased between 2006 and 2018, indicating an unsynchronized relationship between species diversity and coral cover. The increased species diversity may be due to an increase in bare substrates on degraded reefs, creating space for colonization and coexistence in the early stages of succession (Connell 1978; Bongers et al. 2009; Rozendaal et al. 2019). Most of the recovered species by 2018 were encrusting and massive corals, which promoted the subsequent increase in species diversity and functional redundancy but not in trait diversity. Functional dispersion remained stable here, suggesting that the types of dominant species did not change (Laliberté and Legendre 2010; Mouillot et al. 2013). This indicated that while coral species richness continued to increase, those that proliferated during degradation and slow recovery exhibited similar functional traits.

Studies have shown that species richness can recover faster than species composition and functional trait diversity (Rozendaal et al. 2019; Poorter et al. 2021), including amphibians, reptiles, and birds (Acevedo‐Charry and Aide 2019), but there are also studies with the opposite conclusion (Tölgyesi et al. 2019). The present study found that trait diversity of coral assemblages recovered more slowly than species richness, which is consistent with previous findings in coral species (McWilliam et al. 2020). The changes in the volume of trait space show that the cover of critical coral groups in southern Hainan Island has not recovered to its original level. The loss of functional trait diversity may be related to delayed recovery of key functional groups (Stratford and Stouffer 2015; Acevedo‐Charry and Aide 2019). Some functional groups, such as corymbose coral species (*Acropora valida* and *A. nasuta*) that represent high surface areas and fractal structures (Zawada et al. 2019), were not recovered, causing a lag in the recovery of trait diversity. In the northern Red Sea, corymbose corals also showed a risk of population decline due to the lack of small‐size groups (Kramer et al. 2020), indicating the fragility of these species.

Although rarefaction analysis was used to assess sample coverage (Hsieh, Ma, and Chao 2016), it is important to recognize that differences in transect length and replication among years may still affect the results here. Variations in sampling protocols may lead to inconsistencies in the representation of coral species diversity and abundance. For example, the greater number of transects in 2010 and 2018 may have resulted in more coral species being recorded than in 2006, according to species‐area relationship theory (Conor and McCoy 2013). However, this does not alter the main conclusions of the study. Our results for the same survey area in 2010 and 2018 confirm an increase in species diversity in 2018. Furthermore, our results also indicate a loss of coral trait diversity in 2010 and 2018 compared to 2006, which contradicts the expectation that an increase in survey area would lead to a parallel increase in species (Conor and McCoy 2013) and, consequently, in functional diversity (McWilliam et al. 2018; Wong et al. 2018). This further illustrates the disconnect between species diversity and functional diversity in the coral communities of southern Hainan.

The observed deficits in coral traits during the succession process pose significant challenges to the continued recovery of coral reefs in this region, warranting the protection of key functional groups in recovering reef ecosystems. The recolonization of species usually requires a suitable environment, a lack of strong competitors (e.g., algae), and the production of sufficient propagules and recruits (Cadotte and Tucker 2017; D'Amen et al. 2018). In addition to multiple environmental stresses that may affect the recovery of branched corals here (Luo et al. 2023), the lack of propagules and recruits due to the depletion of mature corymbose corals is also likely to be the reason for their lagging recovery (Wood et al. 2014; Dorman et al. 2016). Therefore, to protect these vulnerable coral traits, the priority should be the protection of surviving corymbose corals, which can be achieved by establishing protected areas to limit human activities (Strain et al. 2019) and reducing terrestrial inputs to improve reef conditions (Luo et al. 2023). Meanwhile, the enhancing larval survival of corymbose corals by improving substrate conditions necessary for larval recruitment is crucial, and this could involve reducing sedimentation on the reef surface and removing benthic biological competitors (Zhang et al. 2018; Tebbett, Connolly, and Bellwood 2023). Furthermore, replenishing traits through targeted coral restoration efforts is vital, particularly as techniques for coral‐based outplanting have become increasingly sophisticated (Boström‐Einarsson et al. 2020; Huang et al. 2023).

### 4.3 The Effects of Coral Cover and Trait Diversity on Reef Fish Abundance

Various factors influence the abundance of reef fish. The present study found that reef fish in southern Hainan Island have become less abundant over time, consistent with previous studies (Huang et al. 2017). The decline in reef fish abundance here was mainly due to a decrease in NCD fish, while there was no significant decrease in CD fish. Since NCD fish are more likely to be captured by humans than CD fish (Abesamis et al. 2014; Russ et al. 2021), it is presumed that the decline in fish abundance here may be caused by continued fishing pressure (Huang et al. 2017). Furthermore, our results showed no significant difference in fish assemblage composition between reserve and non‐reserve areas. This suggests that despite recent improvements in protecting and managing coral reef fish in southern Hainan (Huang et al. 2017; Sun et al. 2022), the challenge of protecting their assemblages remains.

The relationship between the biomass and structural complexity of habitat‐forming species and the diversity and biomass of species inhabiting key habitats is a major topic in biodiversity conservation (Hilje et al. 2020). One of the crucial case studies on this topic is the relationship between coral‐forming structural complexity and the diversity and abundance of reef fish. So far, research has shown mixed results, with some studies suggesting that live coral cover and complexity are the important factors for reef fish diversity and abundance (Wilson et al. 2008; Coker, Wilson, and Pratchett 2014; Richardson et al. 2017; Fontoura et al. 2019; Russ et al. 2021), while others have found a weak relationship between corals and reef fishes (Wismer et al. 2019a, 2019b). Moreover, studies have shown that coral‐dwelling fish are not “specific” to the complexity provided by corals in the reef but are more dependent on the complex geospatial structure of the reef as a whole (Siqueira, Muruga, and Bellwood 2023). This study found that coral cover was positively correlated with large NCD and small CD fish. Despite the impact of fisheries on the abundance of NCD fish not being excluded, those results reflected the positive effects that coral‐created habitats on coral fish abundance. Similarly, the trait diversity of habitat‐forming corals was marginally positively correlated with the abundance of small CD fish. Because the coral traits selected in the study are important for the formation of reef fish habitats, encompassing habitat complexity and stability (McWilliam et al. 2018; Zawada et al. 2019), this positive correlation implies that increased complexity provided by corals may help to maintain small coral‐dwelling fish biomass (Fontoura et al. 2019; Siqueira, Muruga, and Bellwood 2023).

## 5 Conclusions

Understanding the decline and recovery patterns of reef‐building corals is essential to understanding how coral reefs will function in the future. Our study on the recovery dynamics of reef‐building corals and reef fish assemblages in southern Hainan Island revealed a slow recovery of coral cover after degradation. However, the recovery of coral trait diversity was hindered by the lack of recovery of specific coral species, with declines in corymbose corals being the primary cause of the limited recovery of coral trait diversity. This underscores the necessity to conserve vital species and traits. Moreover, reef fish abundance was positively related to coral cover, indicating that the abundance of habitat‐forming taxa is critical for maintaining ecosystem biomass more broadly. These results illustrate the dynamics of coral assemblages' recovery in southern Hainan Island and the key role of coral trait diversity in supporting the biomass of reef fauna.

## Author Contributions

**Lintao Huang:** conceptualization (lead), data curation (lead), methodology (lead), project administration (supporting), resources (equal), software (lead), validation (equal), visualization (lead), writing – original draft (lead), writing – review and editing (equal). **Mike McWilliam:** conceptualization (equal), formal analysis (equal), methodology (equal), software (equal), writing – original draft (equal), writing – review and editing (equal). **Chengyue Liu:** conceptualization (equal), data curation (supporting), investigation (lead), methodology (supporting), supervision (supporting), writing – original draft (equal), writing – review and editing (equal). **Xiaolei Yu:** investigation (equal), methodology (supporting), writing – original draft (equal). **Lei Jiang:** conceptualization (supporting), data curation (supporting), methodology (supporting), resources (supporting), writing – original draft (supporting). **Chen Zhang:** methodology (supporting), validation (supporting), writing – original draft (supporting). **Yong Luo:** investigation (supporting), methodology (supporting), validation (supporting). **Jianhui Yang:** data curation (equal), investigation (equal), resources (equal). **Xiangcheng Yuan:** investigation (supporting), methodology (supporting), resources (equal), validation (supporting). **Jiansheng Lian:** data curation (lead), investigation (equal), methodology (equal), resources (equal), visualization (equal). **Hui Huang:** conceptualization (lead), formal analysis (equal), funding acquisition (lead), methodology (equal), project administration (lead), resources (lead), supervision (lead), writing – original draft (equal), writing – review and editing (lead).

## Conflicts of Interest

The authors declare no conflicts of interest.

## Supporting information


Data S1

## Data Availability

The data associated with the article has been archived in the Science data bank (https://www.scidb.cn/s/2euEvm).
