# Supplementary material for: Loss of Coral Trait Diversity and Impacts on Reef Fish Assemblages on Recovering Reefs
Source: Ecol Evol. 2024 Oct 30;14(11):e70510. doi: 10.1002/ece3.70510 (PMC11522916; doi:10.1002/ece3.70510)
Supplement: Supplementary file 1 — Data S1 [file ECE3-14-e70510-s001.pdf]

Supplementary Information to "Loss of coral trait diversity and impacts on reef fish assemblages on recovering reefs".

Table S1 Species survey of corals and reef fish in Southern Hainan Island

Table S2 Seven coral traits used to calculate the functional trait parameters, and their functional relevance

Table S3 Parameters of the linear mixed models

Table S4 Analysis of variance assessing the site diversity parameter of coral assemblages for each survey year

Table S5 Analysis of variance assessing the coral reefs fish abundance change for each survey year

Figure S1 The coral community composition forming convex hull in the first two axes of trait space for each survey year. a: 2006; b: 2010; c: 2018; d: Seven traits used to construct the principal coordinates analysis, and the trait vectors are skeletal density (SD), surface area-to-volume ratio (SA/V), growth rate (GR), interstitial space size (ISS), colony maximum diameter (CMD), colony height (CH), and corallite width maximum (CWM).

Figure S2 The coral community composition forming convex hull in the 3-4 axes of trait space for each survey year. a: 2006; b: 2010; c: 2018; d: Seven traits used to construct the principal coordinates analysis, and the trait vectors are skeletal density (SD), surface area-to-volume ratio (SA/V), growth rate (GR), interstitial space size (ISS), colony maximum diameter (CMD), colony height (CH), and corallite width maximum (CWM).

Figure S3 Sites diversity parameters of coral communities in Southern Hainan Island. a: Species richness; b: Trait diversity; c: The average sum of nearest neighbor distances for the nearest five species showing the functional redundancy; d: Functional dispersion. The ANOVA results showed that all parameters were not significantly different between years.

Figure S4 Sample coverage and species diversity estimation of reef fish communities among years. (A) Sample coverage estimation of coral communities among years. (B) Species diversity estimation of the different q orders. When $q = 0$: Species richness; $q = 1$: Shannon diversity; $q = 2$: Simpson diversity. The solid shape marks the observed diversity values.

Figure S5 Distance-based redundancy analysis showed the relationship between reef fish assemblages and abundance of coral reef fishes with different body lengths and ecological characteristics (Coral dwelling fish or CD and Non-coral dwelling fish or NCD). Color showed survey years and point shape showed protected status. Y represented protected reef areas and N represented unprotected reef areas.

Figure S6 Reef fish abundance among survey years. a: Total reef fish abundance; b: Coral dwelling fish abundance; c: non-coral dwelling fish abundance. Different letters within figure indicated significant differences ($p < 0.05$) among years by using LSD multiple comparisons.

Table S1 Species survey of corals and reef fish in Southern Hainan Island

Survey Species	Year	Survey Sites	Protected area or not	Survey Method	Number of Survey Depth	Survey size per Depth	Number of Replicated per Depth
Coral	2006	XD1	Non-Protected	Line intercept transect surveys	3m and 6m	50m	1
		XD2	Protected	Line intercept transect surveys	6m	50m	1
		DD	Protected	Line intercept transect surveys	3m and 6m	50m	1
		LHT1	Protected	Line intercept transect surveys	3m and 6m	50m	1
		LHT2	Non-Protected	Line intercept transect surveys	6m	50m	1
		XDH	Non-Protected	Line intercept transect surveys	3m and 6m	50m	1
		DP	Non-Protected	Line intercept transect surveys	3m and 6m	50m	1
		XP	Protected	Line intercept transect surveys	3m and 6m	50m	1
	2010	XD1	Non-Protected	Line intercept transect surveys	3m and 6m	10m	6
		XD2	Protected	Line intercept transect surveys	6m	10m	6
		DD	Protected	Line intercept transect surveys	3m and 6m	10m	6
		LHT1	Protected	Line intercept transect surveys	3m and 6m	10m	6
		LHT2	Non-Protected	Line intercept transect surveys	6m	10m	6
		XDH	Non-Protected	Line intercept transect surveys	3m and 6m	10m	6
		DP	Non-Protected	Line intercept transect surveys	3m and 6m	10m	6
		XP	Protected	Line intercept transect surveys	3m and 6m	10m	6
	2018	XD1	Non-Protected	Line intercept transect surveys	3m and 6m	10m	6
		XD2	Protected	Line intercept transect surveys	6m	10m	6
		DD	Protected	Line intercept transect surveys	3m and 6m	10m	6
		LHT1	Protected	Line intercept transect surveys	3m and 6m	10m	6
		LHT2	Non-Protected	Line intercept transect surveys	6m	10m	6
		XDH	Non-Protected	Line intercept transect surveys	3m and 6m	10m	6
		DP	Non-Protected	Line intercept transect surveys	3m and 6m	10m	6
		XP	Protected	Line intercept transect surveys	3m and 6m	10m	6
Reef fish	2006	XD2	Protected	Visual transect surveys	5m and 8m	60m^2	5
		DD	Protected	Visual transect surveys	3m and 5m	60m^2	5
		LHT1	Protected	Visual transect surveys	3m	60m^2	5
		XDH	Non-Protected	Visual transect surveys	3m and 5m	60m^2	5
		DP	Non-Protected	Visual transect surveys	3m and 5m	60m^2	5
	2010	XD2	Protected	Visual transect surveys	3m, 6m and 9m	60m^2	5
		DD	Protected	Visual transect surveys	6m and 9m	60m^2	5
		LHT1	Protected	Visual transect surveys	2m and 4m	60m^2	5
		XDH	Non-Protected	Visual transect surveys	3m, 6m and 9m	60m^2	5
		DP	Non-Protected	Visual transect surveys	3m, 6m and 9m	60m^2	5
	2018	XD2	Protected	Visual transect surveys	3m and 6m	40m^2	3
		DD	Protected	Visual transect surveys	3m and 6m	40m^2	3
		LHT1	Protected	Visual transect surveys	3m and 6m	40m^2	3
		XDH	Non-Protected	Visual transect surveys	3m and 6m	40m^2	3
		DP	Non-Protected	Visual transect surveys	3m and 6m	40m^2	3

Table S2 Seven coral traits used to calculate the functional trait parameters, and their functional relevance

Trait	Categories used	Reef function
Growth rate	0–5 (1), 5–10 (2), 10–25 (3), 25–50 (4), and 50–200 (5) mm·y^{-1}	Carbonate framework accretion, reef regeneration
Skeletal density	0–1.2 (1), 1.2–1.5 (2), 1.5–1.8 (3), 1.8–2.1 (4), and 2.1–3 (5) g/cm^3	Carbonate framework accretion
Maximum colony size (diameter)	0–50 (1), 50–100 (2), 100–200 (3), 200–400 (4), and 400–2,000 (5) cm	Carbonate framework accretion, habitat provision and productivity
Corallite width	0–1.5 (1), 1.5–6 (2), 6–12 (3), 12–25 (4), and 25–100 (5) mm	Filter feeding, nutrient capture
Interstitial space size	(1–5) based on morphological categories	Habitat provision
Colony height	(1–5) based on morphological categories	Carbonate framework accretion, habitat provision
Surface area-to-volume ratio	(1–5) based on morphological categories	Primary productivity, nutrient cycling

Table S3 Parameters of the linear mixed models

<table>
<tr><th>Fixed effects</th><th>Estimate</th><th>Std.Error</th><th>df</th><th>t value</th><th>Pr(>|t|)</th><th>Random effects</th><th>Variance</th><th>SD</th></tr>
<tr><td colspan="9">Coral Cover ~ Conservation Status + Times + (1 | Sites)</td></tr>
<tr><td>(Intercept)</td><td>-0.2332</td><td>0.0693</td><td>13</td><td>-3.3640</td><td>0.0051**</td><td>Site (Intercept)</td><td>0.00</td><td>0.00</td></tr>
<tr><td>Conservation Status（Protected）</td><td>0.1975</td><td>0.0801</td><td>13</td><td>2.4670</td><td>0.0283*</td><td></td><td></td><td></td></tr>
<tr><td>Times (2018 - 2010)</td><td>0.2314</td><td>0.0801</td><td>13</td><td>2.8900</td><td>0.0126*</td><td></td><td></td><td></td></tr>
<tr><td colspan="9">Total reef fish abundance (<5cm) ~ Coral functional trait diversity + Coral Cover + (1 | Sites) + (1 | Survey Years)</td></tr>
<tr><td>(Intercept)</td><td>1.9019</td><td>2.1447</td><td>6.7705</td><td>0.8870</td><td>0.4060</td><td>Site (Intercept)</td><td>2.37</td><td>1.54</td></tr>
<tr><td>Coral functional trait diversity</td><td>2.4327</td><td>2.6650</td><td>10.2084</td><td>0.9130</td><td>0.3820</td><td>Year (Intercept)</td><td>5.00</td><td>2.24</td></tr>
<tr><td>Coral Cover</td><td>0.7194</td><td>3.6798</td><td>7.7149</td><td>0.1960</td><td>0.8500</td><td></td><td></td><td></td></tr>
<tr><td colspan="9">Total reef fish abundance (5-10cm) ~ Coral functional trait diversity + Coral Cover + (1 | Sites) + (1 | Survey Years)</td></tr>
<tr><td>(Intercept)</td><td>20.6100</td><td>22.0470</td><td>9.464</td><td>0.9350</td><td>0.3730</td><td>Site (Intercept)</td><td>333.10</td><td>18.25</td></tr>
<tr><td>Coral functional trait diversity</td><td>7.2030</td><td>30.0060</td><td>11.004</td><td>0.2400</td><td>0.8150</td><td>Year (Intercept)</td><td>337.30</td><td>18.37</td></tr>
<tr><td>Coral Cover</td><td>24.7150</td><td>40.9410</td><td>9.957</td><td>0.6040</td><td>0.5600</td><td></td><td></td><td></td></tr>
<tr><td colspan="9">Total reef fish abundance (10-20cm) ~ Coral functional trait diversity + Coral Cover + (1 | Sites) + (1 | Survey Years)</td></tr>
<tr><td>(Intercept)</td><td>0.7644</td><td>1.9449</td><td>9.1389</td><td>0.3930</td><td>0.7030</td><td>Site (Intercept)</td><td>0.00</td><td>0.00</td></tr>
<tr><td>Coral functional trait diversity</td><td>1.5413</td><td>2.9255</td><td>11.8462</td><td>0.5270</td><td>0.6080</td><td>Year (Intercept)</td><td>0.35</td><td>0.60</td></tr>
<tr><td>Coral Cover</td><td>1.3728</td><td>4.2887</td><td>11.5556</td><td>0.3200</td><td>0.7550</td><td></td><td></td><td></td></tr>
<tr><td colspan="9">Total reef fish abundance (>20cm) ~ Coral functional trait diversity + Coral Cover + (1 | Sites) + (1 | Survey Years)</td></tr>
<tr><td>(Intercept)</td><td>-0.0387</td><td>0.1407</td><td>9.64106</td><td>-0.2750</td><td>0.7890</td><td>Site (Intercept)</td><td>0.01</td><td>0.09</td></tr>
<tr><td>Coral functional trait diversity</td><td>-0.0509</td><td>0.1987</td><td>10.70221</td><td>-0.2560</td><td>0.8030</td><td>Year (Intercept)</td><td>0.01</td><td>0.10</td></tr>
<tr><td>Coral Cover</td><td>0.6642</td><td>0.2801</td><td>11.0393</td><td>2.3710</td><td>0.0370*</td><td></td><td></td><td></td></tr>
<tr><td colspan="9">Coral dwelling reef fish abundance (<5cm) ~ Coral functional trait diversity + Coral Cover + (1 | Sites) + (1 | Survey Years)</td></tr>
<tr><td>(Intercept)</td><td>-0.8371</td><td>0.9353</td><td>12</td><td>-0.8950</td><td>0.3884</td><td>Site (Intercept)</td><td>0.00</td><td>0.00</td></tr>
<tr><td>Coral functional trait diversity</td><td>2.8572</td><td>1.4346</td><td>12</td><td>1.9920</td><td>0.0697·</td><td>Year (Intercept)</td><td>0.00</td><td>0.00</td></tr>
<tr><td>Coral Cover</td><td>2.6927</td><td>2.0823</td><td>12</td><td>1.2930</td><td>0.2203</td><td></td><td></td><td></td></tr>
<tr><td colspan="9">Coral dwelling reef fish abundance (5-10cm) ~ Coral functional trait diversity + Coral Cover + (1 | Sites) + (1 | Survey Years)</td></tr>
<tr><td>(Intercept)</td><td>-0.9934</td><td>8.3527</td><td>8.7704</td><td>-0.1190</td><td>0.9080</td><td>Site (Intercept)</td><td>333.10</td><td>18.25</td></tr>
<tr><td>Coral functional trait diversity</td><td>2.5107</td><td>8.6873</td><td>7.6168</td><td>0.2890</td><td>0.7803</td><td>Year (Intercept)</td><td>337.30</td><td>18.37</td></tr>
<tr><td>Coral Cover</td><td>49.1937</td><td>11.1203</td><td>7.1481</td><td>4.4240</td><td>0.0029**</td><td></td><td></td><td></td></tr>
<tr><td colspan="9">Coral dwelling reef fish abundance (10-20cm) ~ Coral functional trait diversity + Coral Cover + (1 | Sites) + (1 | Survey Years)</td></tr>
<tr><td>(Intercept)</td><td>-0.6596</td><td>1.4271</td><td>12</td><td>-0.4620</td><td>0.6520</td><td>Site (Intercept)</td><td>0.00</td><td>0.00</td></tr>
<tr><td>Coral functional trait diversity</td><td>2.4538</td><td>2.1891</td><td>12</td><td>1.1210</td><td>0.2840</td><td>Year (Intercept)</td><td>0.00</td><td>0.00</td></tr>
<tr><td>Coral Cover</td><td>-0.1099</td><td>3.1773</td><td>12</td><td>-0.0350</td><td>0.9730</td><td></td><td></td><td></td></tr>
</table>

Coral dwelling reef fish abundance (>20cm) ~ Coral functional trait diversity + Coral Cover + (1 | Sites) + (1 | Survey Years)								
(Intercept)	0.0187	0.0231	9.11656	0.8080	0.4390	Site (Intercept)	0.00	0.00
Coral functional trait diversity	0.0271	0.0334	11.42415	0.8120	0.4330	Year (Intercept)	0.00	0.01
Coral Cover	-0.0686	0.0497	11.99629	-1.3800	0.1930			
Non-coral dwelling reef fish abundance (<5cm) ~ Coral functional trait diversity + Coral Cover + (1 | Sites) + (1 | Survey Years)								
(Intercept)	1.9040	1.3500	6.981	1.4110	0.2010	Site (Intercept)	0.00	0.00
Coral functional trait diversity	-1.2590	1.6900	10.783	-0.7450	0.4720	Year (Intercept)	1.75	1.32
Coral Cover	2.4700	2.6350	11.265	0.9380	0.3680			
Non-coral dwelling reef fish abundance (5-10cm) ~ Coral functional trait diversity + Coral Cover + (1 | Sites) + (1 | Survey Years)								
(Intercept)	21.9920	15.1780	6.865	1.4490	0.1910	Site (Intercept)	0.00	0.00
Coral functional trait diversity	6.1620	18.7210	10.771	0.3290	0.7480	Year (Intercept)	234.60	15.32
Coral Cover	-28.1470	29.2080	11.219	-0.9640	0.3560			
Non-coral dwelling reef fish abundance (10-20cm) ~ Coral functional trait diversity + Coral Cover + (1 | Sites) + (1 | Survey Years)								
(Intercept)	1.1477	1.1740	11.7087	0.9780	0.3480	Site (Intercept)	0.32	0.56
Coral functional trait diversity	-0.5955	1.8250	11.1492	-0.3260	0.7500	Year (Intercept)	0.00	0.00
Coral Cover	1.8616	2.6542	11.5867	0.7010	0.4970			
Non-coral dwelling reef fish abundance (>20cm) ~ Coral functional trait diversity + Coral Cover + (1 | Sites) + (1 | Survey Years)								
(Intercept)	-0.0353	0.1275	9.92802	-0.2770	0.7875	Site (Intercept)	0.01	0.12
Coral functional trait diversity	-0.1282	0.1815	11.61148	-0.7060	0.4939	Year (Intercept)	0.01	0.09
Coral Cover	0.7363	0.2510	10.2213	2.9340	**0.0146***			

Note: · p<0.1, * p<0.05; df: the Degrees of Freedom; Conservation Status (Protected) indicated sites within the MPA; Times (2018 - 2010) indicated coral cover in 2018 minus cover in 2010; The italics showed the functions for the linear mixed model.

Table S4 Analysis of variance assessing the site diversity parameter of coral assemblages for each survey year

**Variables**	**SS**	***df***	**MS**	***F value***	***P***
Species richness	397.6	2	198.79	2.117	0.145
Trait diversity	0.2258	2	0.1129	1.683	0.21
Functional redundancy	0.4160	2	0.2082	1.245	0.308
Functional dispersion	0.0181	2	0.0091	1.485	0.249

Note: SS: Sum of Squares; df: the Degrees of Freedom; MS: the Mean Square.

Table S5 Analysis of variance assessing the coral reefs fish abundance change for each survey year

**Variables**	**SS**	***df***	**MS**	***F value***	***P***
Total reef fish abundance	4695	2	2347	3.166	**0.0787**$^{\cdot}$
Coral-dwelling reef fish abundance	289.9	2	145	0.665	0.532
Non coral dwelling reef fish abundance	2695	2	1347.6	4.811	**0.0292***

Note: $^{\cdot}$ $p<0.1$, * $p<0.05$; SS: Sum of Squares; df: the Degrees of Freedom; MS: the Mean Square.

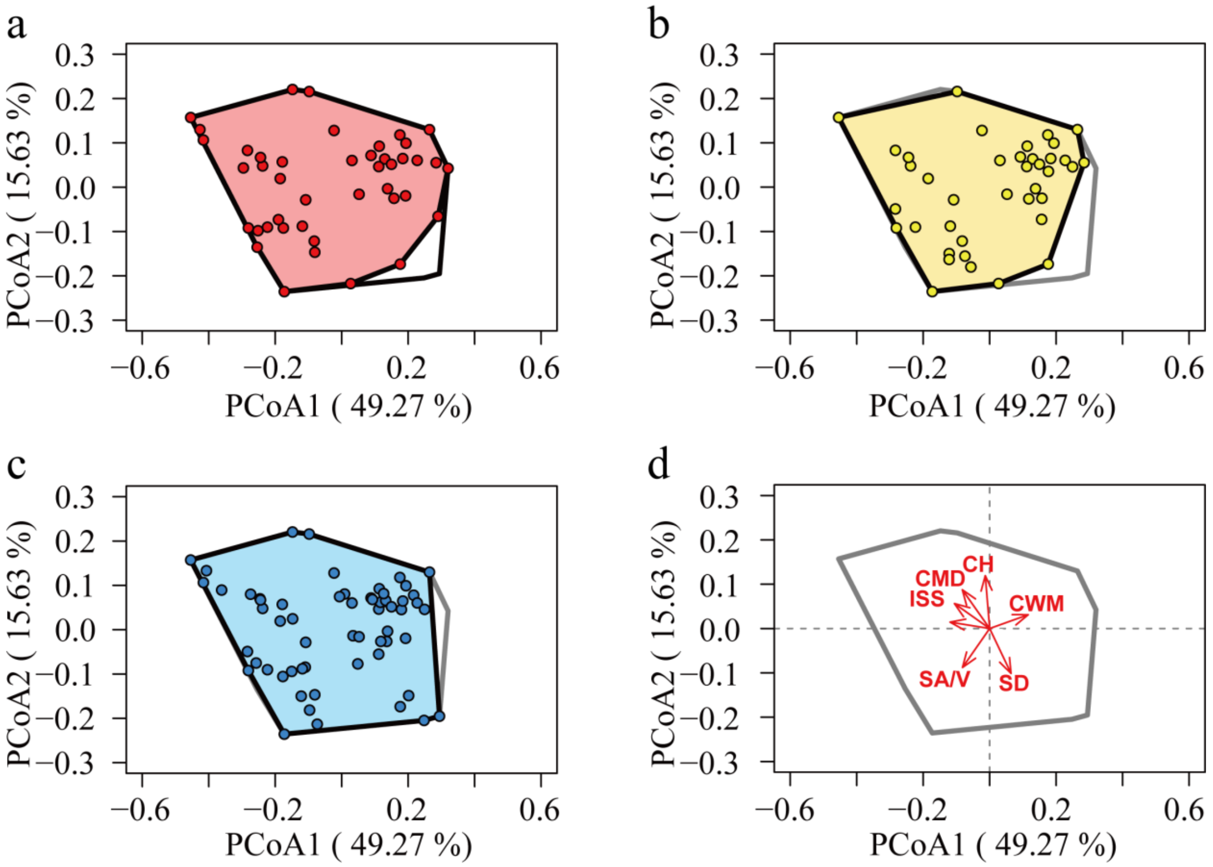

Figure S1 The coral community composition forming convex hull in the first two axes of trait space for each survey year. a: 2006; b: 2010; c: 2018; d: Seven traits used to construct the principal coordinates analysis, and the trait vectors are skeletal density (SD), surface area-to-volume ratio (SA/V), growth rate (GR), interstitial space size (ISS), colony maximum diameter (CMD), colony height (CH), and corallite width maximum (CWM).

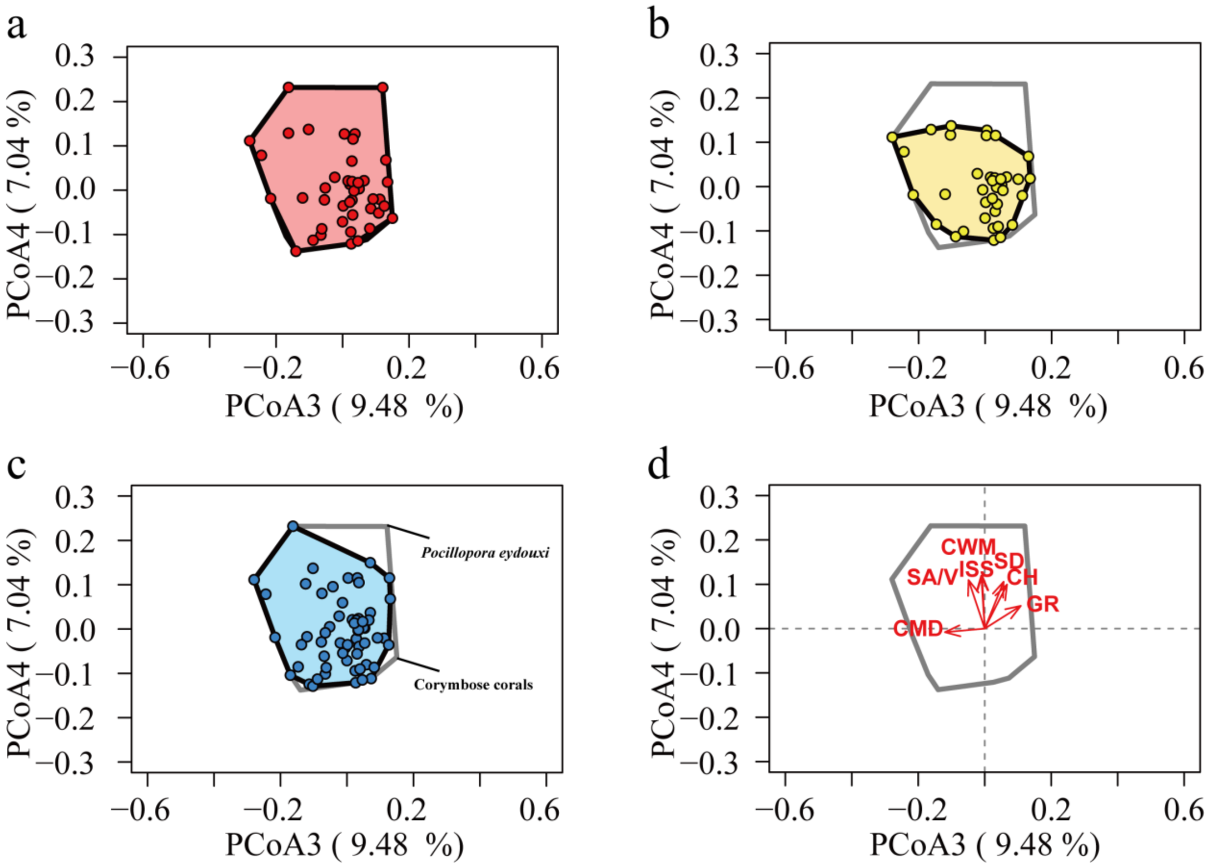

Figure S2 The coral community composition forming convex hull in the 3-4 axes of trait space for each survey year. a: 2006; b: 2010; c: 2018; d: Seven traits used to construct the principal coordinates analysis, and the trait vectors are skeletal density (SD), surface area-to-volume ratio (SA/V), growth rate (GR), interstitial space size (ISS), colony maximum diameter (CMD), colony height (CH), and corallite width maximum (CWM).

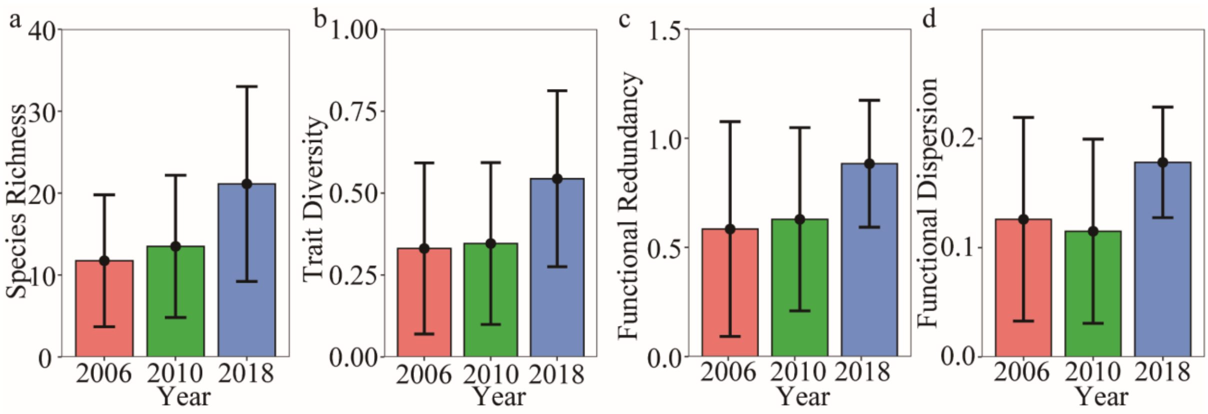

Figure S3 Sites diversity parameters of coral communities in Southern Hainan Island. a: Species richness; b: Functional trait diversity; c: The average sum of nearest neighbor distances for the nearest five species showing the functional redundancy; d: Functional dispersion. The ANOVA results showed that all parameters were not significantly different between years.

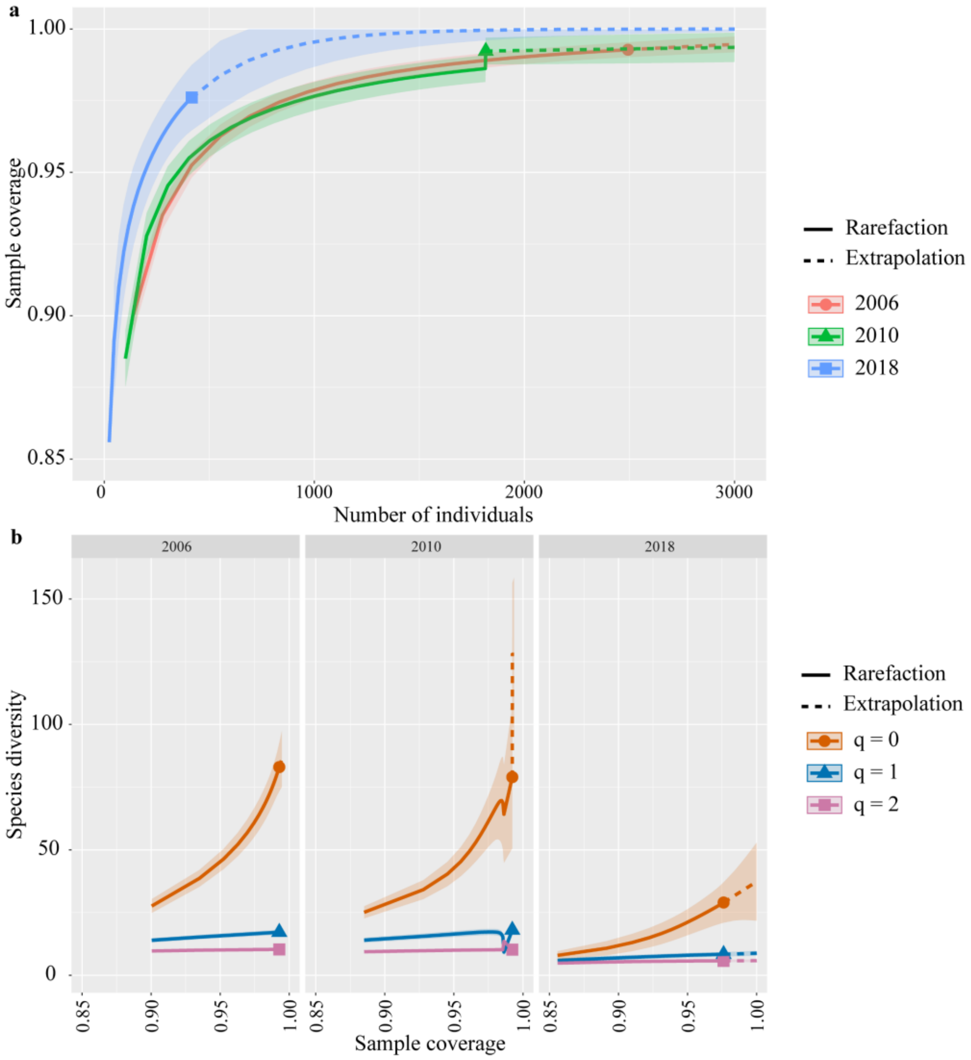

Figure S4 Sample coverage and species diversity estimation of reef fish assemblages among years. (A) Sample coverage estimation of coral communities among years. (B) Species divers

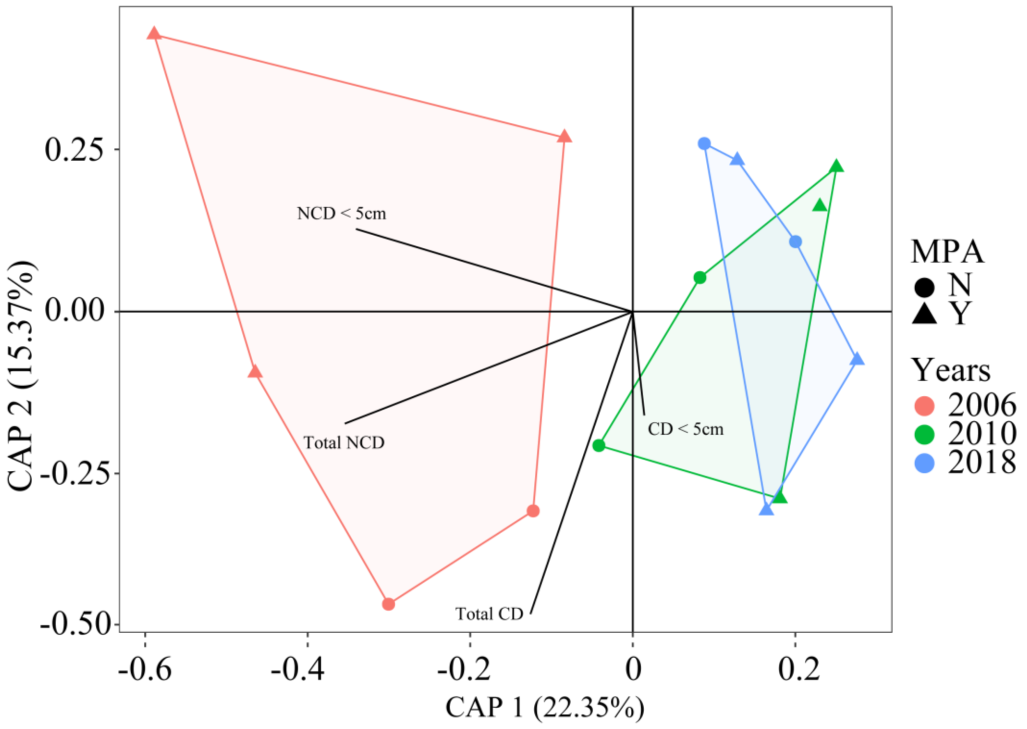

Figure S5 Distance-based redundancy analysis showed the relationship between reef fish assemblages and abundance of coral reef fishes with different body lengths and ecological characteristics (Coral dwelling fish or CD and Non-coral dwelling fish or NCD). Color showed survey years and point shape showed protected status. Y represented protected reef areas and N represented unprotected reef areas.

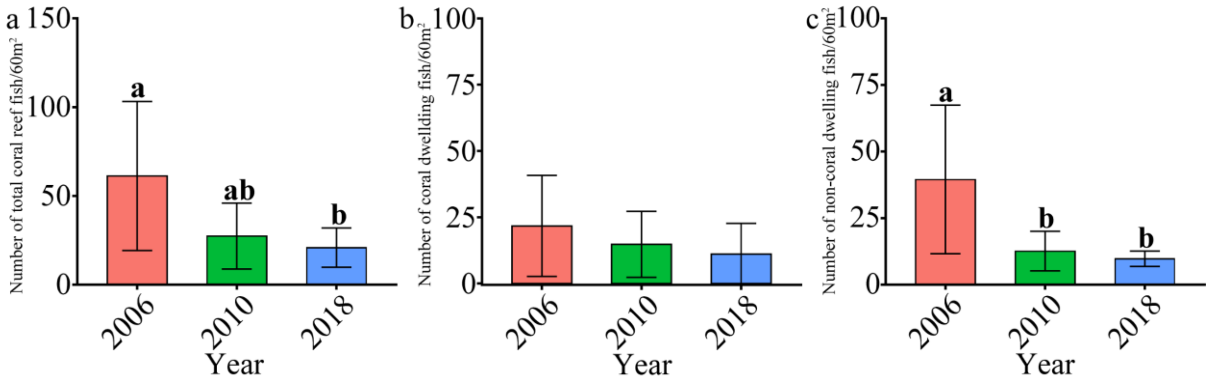

Figure S6 Reef fish abundance among survey years. a: Total reef fish abundance; b: Coral dwelling fish abundance; c: non-coral dwelling fish abundance. Different letters within figure indicated significant differences ($p < 0.05$) among years by using LSD multiple comparisons.
